# Polymer-Assisted Synthesis of Co_3_O_4_ Spinel Catalysts with Enhanced Surface Co^2+^ Ions for N_2_O Decomposition

**DOI:** 10.3390/nano15211642

**Published:** 2025-10-28

**Authors:** Nahea Kim, Su-Jin Kim, Sang-Hyeok Seo, Myeung-Jin Lee, Bora Jeong, Hong-Dae Kim, Tae Won Nam, Bora Ye

**Affiliations:** 1 Ulsan Technology Application Division, Korea Institute of Industrial Technology, Ulsan 44413, Republic of Korea; nahea007@kitech.re.kr (N.K.); sujini@kitech.re.kr (S.-J.K.); seosang0422@kitech.re.kr (S.-H.S.); leemj@kitech.re.kr (M.-J.L.);; 2 Department of Materials Science and Engineering, Pusan National University, Busan 46241, Republic of Korea

**Keywords:** Nitrous oxide decomposition, Co_3_O_4_ spinel catalyst, surface Co^2+^ species

## Abstract

Nitrous oxide (N_2_O) is a potent greenhouse gas with a global warming potential > 310 times that of CO_2_. Owing to its rapid increase in atmospheric concentrations from industrial emissions, N_2_O poses increasing environmental concerns. Among the various N_2_O abatement technologies, catalytic decomposition can directly convert N_2_O into harmless N_2_ and O_2_ without generating secondary pollutants. In this study, Co_3_O_4_ spinel catalysts were synthesized using a polymer-assisted precipitation method, using polyvinyl alcohol, polyvinylpyrrolidone, or polyethylene glycol (PEG) as N_2_O decomposition catalysts. The PEG-mediated synthesis method yielded the most active catalyst with superior N_2_O decomposition efficiency. Structural and surface analyses confirmed that PEG facilitated the formation of Co^2+^-enriched surface sites and abundant oxygen vacancies, which are crucial active sites for N_2_O adsorption and activation. Moreover, these features improved the redox properties and electron transfer behavior of the resulting catalyst. In particular, the PEG-derived 5Co_3_O_4_/CeO_2_ catalyst exhibited enhanced N_2_O decomposition activity and stability even in the presence of coexisting N_2_O and O_2_, highlighting its potential for real-world applications. This study provides an effective synthetic route for Co_3_O_4_-based catalysts and potential opportunities for wide applications in industrial N_2_O removal.

## 1. Introduction

Nitrous oxide (N_2_O) has recently surpassed chlorofluorocarbons (CFCs) as the predominant anthropogenic ozone-depleting substance and is the third most significant greenhouse gas following CO_2_ and CH_4_ [1,2,3]. Although N_2_O represents a relatively small fraction of total greenhouse gas emissions, its global warming potential is approximately 310 times higher than that of CO_2_, and its ozone depletion potential is nearly double that of CFCs [4,5]. The continuous increase in atmospheric N_2_O concentrations caused by industrial activities, including nitric and adipic acid production, semiconductor manufacturing, fossil fuel combustion, co-firing power generation, and maritime emissions, has become an increasingly serious environmental concern [6,7,8]. N_2_O emissions are expected to double by 2050, rendering the development and implementation of effective reduction strategies urgently required. Various mitigation strategies have been developed, including thermal decomposition and catalytic reduction [9,10]. Among them, the direct catalytic decomposition of N_2_O into harmless N_2_ and O_2_ is a promising approach owing to the absence of secondary pollutants and its simplicity and high efficiency [11,12]. For the direct catalytic decomposition of N_2_O, extensive research has focused on three primary catalyst categories: supported noble metals [13,14,15], pure and composite metal oxides [11,16,17,18], and zeolite-based systems [19,20,21]. Among these, metal oxide catalysts with excellent thermal stability at high temperatures are being researched as an alternative to overcome disadvantages such as the high cost of noble metal catalysts, low hydrothermal stability, and catalyst activity degradation by other gas components of zeolite catalysts [22,23]. In particular, spinel-type Co_3_O_4_ (AB_2_O_4_), in which Co^2+^ occupies tetrahedral (A) sites and Co^3+^ occupies octahedral (B) sites, has been widely investigated for N_2_O decomposition. Spinel-type Co_3_O_4_ possesses the favorable redox properties of Co^2+^ and the high density of oxygen vacancies [24,25]. Although Co_3_O_4_-based catalysts offer advantages for N_2_O decomposition, they often exhibit reduced activity in the presence of interfering gases, such as O_2_. These interfering gases compete with N_2_O for active sites and hinder the reaction by suppressing the role of surface oxygen vacancies [26,27]. While several synthesis strategies, such as doping and defect engineering, have been explored for tuning the Co^3+^/Co^2+^ ratio, these methods often entail complex procedures or harsh, high-temperature post-treatments [28,29]. In contrast, the polymer-assisted synthesis offers a facile, energy-efficient, and cost-effective route to concurrently modulate the surface electronic structure and active sites [30]. This is achieved through the coordination between polymer functional groups (e.g., PVA, PVP, PEG) and cobalt precursors, which enables the precise control of the Co^3+^/Co^2+^ ratio and oxygen-vacancy formation under mild reaction conditions, without requiring additional doping or post-treatment [31,32].

To address these limitations, polyvinyl alcohol (PVA), polyvinylpyrrolidone (PVP), and polyethylene glycol (PEG) have been employed as polymeric additives owing to their distinct physicochemical properties. For example, PVA exhibits mild reducing properties [33,34], and PVP stabilizes particle dispersion [34,35]. PEG provides strong coordination capability and promotes the formation of surface-active sites and oxygen vacancies. In particular, PEG has emerged as an effective modifier, achieving the highest N_2_O conversion owing to its abundant hydroxyl groups and flexible molecular backbone, which facilitate enhanced interactions with metal precursors [36,37,38]. Recent density functional theory calculations have demonstrated that the polymer-induced electronic modulation of the surface cobalt oxidation states, coupled with the generation of oxygen vacancies, can effectively decrease the activation energy for N_2_O decomposition [39,40]. Although polymer-assisted synthesis is generally used for morphological control or particle stabilization, its potential role in modulating the surface electronic properties, particularly in oxidative environments, has not been extensively investigated.

In this study, PVA, PVP, and PEG were employed as polymeric additives to modulate the surface properties and electronic structures of Co_3_O_4_ catalysts. To evaluate their catalytic performance, Co_3_O_4_ catalysts, which were synthesized using polymer additives, were supported on CeO_2_ to form 5Co_3_O_4_/CeO_2_ composite catalysts. CeO_2_ was selected because it is widely known for its high oxygen storage capacity and strong redox ability, which promote the formation of surface oxygen vacancies and enhance oxygen mobility [36]. By varying the polymer additives used during synthesis, the Co^2+^/Co^3+^ ratio was successfully tuned and abundant surface oxygen vacancies were introduced, enhancing the N_2_O decomposition activity. The polymer-assisted strategy enabled the formation of Co_3_O_4_ surfaces rich in Co^2+^ and oxygen vacancies, achieving enhanced catalytic activity even under high gas hourly space velocity (GHSV) conditions. Notably, the optimized PEG catalyst maintained over 98% N_2_O conversion above 550 °C even under oxygen-rich conditions, further indicating its potential applicability in realistic industrial environments.

## 2. Materials and Methods

### 2.1. Materials

Cobalt(II) nitrate hexahydrate (Co(NO_3_)_2_·6H_2_O) and cerium(IV) oxide (CeO_2_) were purchased from Sigma-Aldrich (St. Louis, MO, USA). PVA [CH_2_CH(OH)n], PVP [(C_6_H_9_NO)n], PEG [H((CH_2_CH_2_O)n)OH], and sodium hydroxide (NaOH) were obtained from Daejung Chemicals and Metals Co., Ltd. (Siheung-si, Gyeonggi-do, Republic of Korea).

### 2.2. Preparation of Polymer-Assisted Co_3_O_4_ Spinel Catalysts Using PVA, PVP, and PEG

Polymer-assisted Co_3_O_4_ spinel catalysts were synthesized using a precipitation method. Briefly, 1.0 and 10.0 g of polymer and NaOH, respectively, were dissolved in 100 mL of deionized (DI) water and stirred for 1 h. Subsequently, 15.0 g of Co(NO_3_)_2_·6H_2_O was dissolved in 100 mL of DI water and stirred for 1 h. A mixed solution of the polymer and NaOH was slowly added dropwise to the prepared cobalt solution. The pink precipitate formed from the initial blue solution was washed with ethanol and DI water and repeatedly centrifuged until the pH became neutral. Subsequently, the catalyst precursors were dried at 100 °C for 6 h in an oven and calcined at 650 °C for 4 h in a muffle furnace. The synthesized PEG-, PVP-, and PVA-based catalysts were labeled as PEG Co_3_O_4_, PVP Co_3_O_4_, and PVA Co_3_O_4_, respectively.

### 2.3. Synthesis of Polymer-Assisted Co_3_O_4_/CeO_2_ Catalysts

Using the impregnation method, the polymer-assisted (PVA, PVP, or PEG) catalysts were used as precursors to obtain CeO_2_-supported catalysts (5Co_3_O_4_/CeO_2_). Briefly, 5 wt% polymer-assisted (PVA, PVP, or PEG) Co_3_O_4_ was stirred in 100 mL of DI water for 30 min, while CeO_2_ was stirred in 150 mL of DI water for 30 min. The solutions were mixed together and stirred for 1 h. The catalysts were dried at 100 °C for 6 h in an oven and calcined at 650 °C for 4 h in a muffle furnace. The PEG-, PVP-, and PVA-assisted/CeO_2_-supported catalysts were labeled as PEG 5Co_3_O_4_/CeO_2_, PVP 5Co_3_O_4_/CeO_2_, and PVA 5Co_3_O_4_/CeO_2_, respectively. To enhance visual clarity and comprehensibility, the overall synthesis route has been illustrated as a schematic representation, which has been incorporated into the Figure S1.

### 2.4. Catalyst Characterization

The structural characteristics of the catalysts were analyzed using X-Ray diffraction (XRD; D8 Advance, Bruker, Billerica, MA, USA) with Cu Kα radiation over a 2θ range of 10–80° to confirm the formation of the spinel Co_3_O_4_ phase and to evaluate the effect of polymer additives on crystallinity. The morphology and lattice structure of the catalysts were examined using field emission transmission electron microscopy (JEM-F200, JEOL, Ltd., Tokyo, Japan) operated at 200 kV. The specific surface areas and pore volumes of the catalysts were determined using the Brunauer–Emmett–Teller (BET) and Barrett–Joyner–Halenda methods with an ASAP 2020 instrument (Micromeritics, Norcross, GA, USA) to assess the influence of polymer-assisted synthesis on textural properties. Prior to obtaining measurements, the samples were pretreated at 150 °C for 4 h under vacuum with N_2_ gas to remove impurities and moisture. Bonding between the polymer and metal ion state (Co^2+^ and Co^3+^) was evaluated using Fourier-transform infrared (FT-IR) spectroscopy (Vertex 80v, Bruker, USA). The presence of residual polymer was confirmed using a micro-Raman spectrometer (NRS-5100, JASCO, Tokyo, Japan) with a laser wavelength of 532 nm. X-Ray photoelectron spectroscopy (XPS; K-Alpha, Thermo Fisher Scientific, Waltham, MA, USA) using an Al Kα radiation source (λ = 1.4866 Å) was conducted to investigate the chemical states of elements in the polymer-assisted Co_3_O_4_/CeO_2_ catalyst. The XPS binding energy was calibrated using the C 1s peak at 284.6 eV. Electron paramagnetic resonance (EPR) spectroscopy (Bruker, Billerica, MA, USA, EMXplus) was performed at X-band ~9.518 GHz with a magnetic field 1500 G to measure the unpaired electron signals of Co^2+^ ions for identification and quantification. Temperature-programmed reduction with H_2_ (H_2_-TPR) and temperature-programmed desorption with O_2_ (O_2_-TPD) and N_2_O (N_2_O-TPD) were performed to evaluate the redox behavior, oxygen mobility, using a chemisorption analyzer (AutoChem II 2920, Micromeritics, USA) by increasing the temperature from 100 to 700 °C at a rate of 10 °C/min.

### 2.5. Catalytic Performance Evaluation

The N_2_O decomposition activity was evaluated at 350–650 °C using a fixed-bed reactor with a reactor diameter and length of 12.7 and 400 mm, respectively. The feed-gas flow rate was regulated using a mass-flow controller (Brooks Instruments, Hatfield, PA, USA). The total flow rate of the feed gas was maintained at 500 sccm. For each test, 0.9 g of catalyst was loaded onto quartz wool 0.3 g to prevent channeling and maintain a stable fixed-bed configuration during the reaction. The evaluation gas flow conditions were as follows: 5000 ppm of N_2_O, 5 vol% O_2_ (when used), balanced N_2_, and GHSV of 60,000 h^−1^. FT-IR spectroscopy (CX-4000, Gasmet Technologies Oy, Helsinki, Finland) was employed to analyze the inlet and outlet reaction gas concentrations (except O_2_), while the O_2_ concentration was measured using a gas analysis spectrometer (DSM-XT, Hausnet S.R.L., Buenos Aires, Argentina). The N_2_O conversion efficiency was calculated using Equation (1), where [N_2_O]_in_ and [N_2_O]_out_ represent the N_2_O concentrations (ppm) in the feed and flue gases, respectively.

(1) $$N_{2}O\;conversion\;(\%)=\frac{{\left[N_{2}O\right]}_{in}-{\left[N_{2}O\right]}_{out}}{{\left[N_{2}O\right]}_{in}}\times 100$$

## 3. Results & Discussion

### 3.1. Catalytic Performance

**Figure 1:**
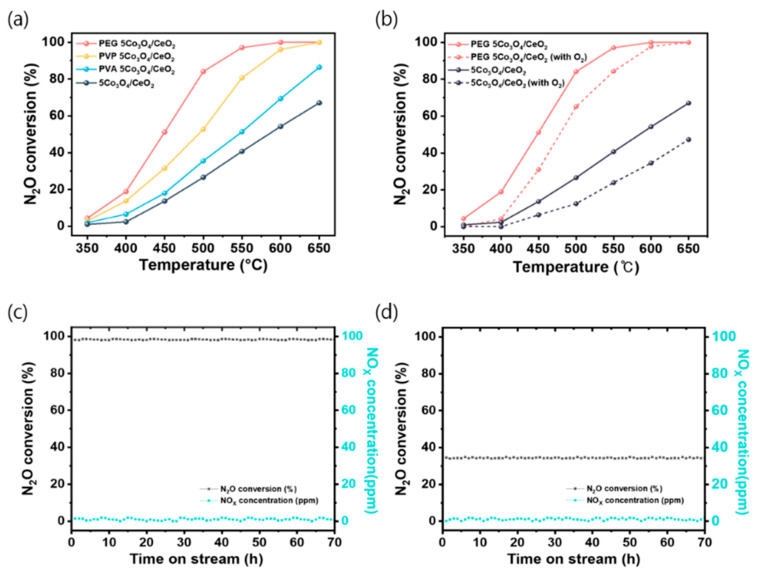
(**a**) N_2_O decomposition activities of the PEG, PVP, and PVA-assisted 5Co_3_O_4_/CeO_2_ catalysts and pristine 5Co_3_O_4_/CeO_2_ under the following conditions: 5000 ppm N_2_O, N_2_ balance, GHSV of 60,000 h^−1^, and 350–650 °C. (**b**) N_2_O decomposition activities of PEG 5Co_3_O_4_/CeO_2_ and pristine 5Co_3_O_4_/CeO_2_ with and without 5 vol.% O_2_ under the following conditions: 5000 ppm N_2_O, N_2_ balance, GHSV of 60,000 h^−1^, and 350–650 °C. Long-term stabilities of the (**c**) PEG 5Co_3_O_4_/CeO_2_ and (**d**) pristine 5Co_3_O_4_/CeO_2_ catalysts at 600 °C for 70 h under the following conditions: 5000 ppm N_2_O, 5 vol.% O_2_, N_2_ balance, and GHSV of 60,000 h^−1^.

The catalytic activity of the synthesized polymer-assisted 5Co_3_O_4_/CeO_2_ catalysts was evaluated for N_2_O decomposition using a fixed-bed reactor. As shown in Figure 1a, all polymer-assisted catalysts exhibited enhanced N_2_O conversion compared to the pristine Co_3_O_4_/CeO_2_. Among them, PEG 5Co_3_O_4_/CeO_2_ demonstrated the highest activity, achieving ˃99.5% conversion at 600 °C. In contrast, the pristine sample reached a maximum conversion of 57.6% under identical conditions. This trend highlights the promotional effect of polymer-induced surface modification, particularly by PEG, on catalytic performance for N_2_O decomposition. The catalytic activity trend observed in Figure S2 for polymer-assisted Co_3_O_4_ catalysts was consistent with that of the corresponding 5Co_3_O_4/_CeO_2_ catalysts. To ensure that the intrinsic catalytic activity was accurately evaluated and to eliminate surface area effects as shown in Figure S3, the N_2_O decomposition reaction was performed under kinetically controlled conditions, where mass-transfer limitations were excluded according to the Koros–Nowak criterion [41,42]. All catalytic measurements were carried out at a GHSV 1,000,000 h^−1^, while maintaining the N_2_O conversion below 20%, consistent with previously reported kinetic control conditions [43]. The apparent activation energies (E_a_) for the Co_3_O_4_ catalysts were determined to be 73.94 kJ∙mol^−1^ for pristine Co_3_O_4_, 59.42 kJ∙mol^−1^ for PVA Co_3_O_4_, kJ∙mol^−1^ for PVP Co_3_O_4_, and kJ∙mol^−1^ for PEG Co_3_O_4_. Significantly, PEG Co_3_O_4_ exhibited the lowest E_a_, indicating that the polymer-assisted precipitation strategy effectively increased the density of accessible active sites and enhanced surface reducibility, thereby improving the intrinsic catalytic activity toward N_2_O decomposition.

To investigate the effect of O_2_ on N_2_O decomposition owing to competitive adsorption on the catalyst surface, catalytic activity under O_2_ co-feeding conditions was evaluated for the best-performing (PEG 5Co_3_O_4_/CeO_2_) and least active (pristine 5Co_3_O_4_/CeO_2_) catalysts [31,44]. As shown in Figure 1b, the presence of 5 vol% O_2_ suppressed N_2_O conversion across all temperatures. The observed inhibitory effect is consistent with previous studies, which have reported that the coexistence of O_2_ suppresses N_2_O decomposition due to competitive adsorption on the active sites of Co_3_O_4_-based catalysts [45,46,47,48]. However, PEG 5Co_3_O_4_/CeO_2_ retained high activity even in the presence of O_2_, reaching 97.6% conversion at 600 °C. In contrast, the pristine 5Co_3_O_4_/CeO_2_ catalyst showed significantly reduced performance, with a maximum conversion decreasing to 35.4% under identical conditions. Thus, PEG 5Co_3_O_4_/CeO_2_ exhibited enhanced intrinsic activity and superior resistance to oxygen-induced inhibition. Long-term stability of the catalysts was evaluated by conducting a 70 h N_2_O decomposition test at 600 °C under O_2_ and N_2_O co-feeding conditions, simulating realistic oxidative environments Figure 1c,d. Because industrial N_2_O abatement systems typically operate under oxidizing atmospheres for extended durations, such long-term O_2_ durability is critical for assessing catalyst viability in commercial-scale industry.

In addition, NOx concentrations in the outlet stream were monitored to assess the selectivity of the catalysts and ensure that N_2_O was directly decomposed into N_2_ and O_2_ without proceeding through intermediate steps involving NO or NO_2_ formation. Monitoring NOx is critical because N_2_O can follow multiple decomposition pathways, some of which involve the formation of NO or NO_2_ as intermediates [1]. These species may arise from incomplete reduction or side oxidation reactions. Their presence indicates both a loss of selectivity toward N_2_ and environmental concerns because NOx are regulated pollutants [49]. Therefore, the absence of NOx formation indicates that the reaction selectively proceeds toward N_2_ and O_2_ without generating harmful byproducts. Under N_2_O and O_2_ conditions, the PEG 5Co_3_O_4_/CeO_2_ catalyst exhibited only a marginal decrease in N_2_O conversion (from ˃99.5% to 97.2%) at 600 °C. In contrast, the pristine 5Co_3_O_4_/CeO_2_ catalyst showed a significant reduction in performance, with the N_2_O conversion decreasing from 57.6% to 35.4%. This reduction represents approximately a 20% loss in activity. Thus, the PEG additive functioned as an effective surface electronic property modulator, enhancing both redox stability and oxygen tolerance. Consequently, PEG 5Co_3_O_4_/CeO_2_ maintained superior catalytic activity even under oxidizing conditions.

**Figure 2:**
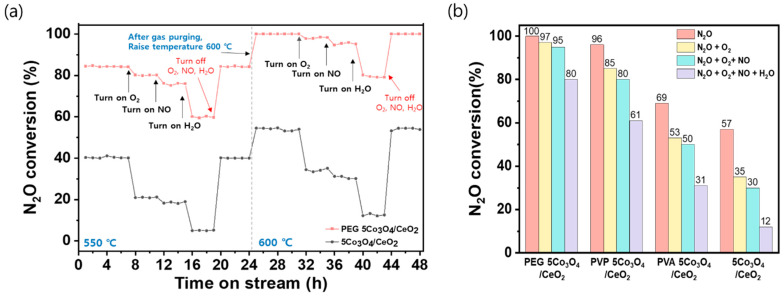
N_2_O decomposition activities of the PEG 5Co_3_O_4_/CeO_2_ and pristine 5Co_3_O_4_/CeO_2_ catalysts under the conditions. (**a**) Time-on-stream stability test at 550 and 600 °C under sequential addition of O_2_, NO, and H_2_O. (**b**) All synthesized catalysts at 600 °C under simultaneous feeding O_2_, NO, and H_2_O. The reaction conditions were as follows: 5000 ppm N_2_O, 5 vol% O_2_, 500 ppm NO, 5 vol% H_2_O, N_2_ balance, and GHSV of 60,000 h^−1^.

To assess the durability of the catalysts under conditions that influence stability, time-on-stream tests were conducted at 550 and 600 °C with the stepwise introduction of O_2_, NO, and H_2_O into the N_2_O feed stream Figure 2a. Furthermore, Figure 2b shows the N_2_O decomposition of all synthesized catalysts at 600 °C under simultaneous feeding conditions. These species adversely affect catalytic performance because O_2_ competes with N_2_O for active sites while NO and H_2_O deactivate or block surface oxygen vacancies, resulting in decreased catalytic activity [50,51]. At 550 °C, the pristine 5Co_3_O_4_/CeO_2_ catalyst exhibited low baseline activity (approximately 40%), which decreased more severely to 21% (with O_2_), 18% (with O_2_ + NO), and only 6% (with O_2_ + NO + H_2_O). In contrast, the PEG 5Co_3_O_4_/CeO_2_ catalyst initially achieved an 84% N_2_O conversion under N_2_O-only conditions. Upon the sequential additions of O_2_, NO, and H_2_O, the conversion progressively decreased to 80%, 76%, and 59%, respectively. A similar trend was observed at 600 °C. The PEG-assisted catalyst maintained ˃99.5% N_2_O conversion under N_2_O-only conditions, with only minor reductions of 97%, 95%, and 80% following co-feeding with O_2_, NO, and H_2_O, respectively. Conversely, the pristine catalyst started at 57% conversion and decreased to 35%, 30%, and 12% under identical conditions. These results demonstrate the superior tolerance of the PEG 5Co_3_O_4_/CeO_2_ catalyst to the deactivation effects of oxygen, nitrogen oxides, and water vapor, which can significantly impair the N_2_O decomposition performance of conventional Co_3_O_4_-based catalysts. Overall, the PEG 5Co_3_O_4_/CeO_2_ catalyst exhibited excellent N_2_O removal efficiency, enhanced resistance to common inhibitors, and stable performance under various reaction conditions. Thus, the PEG 5Co_3_O_4_/CeO_2_ catalyst has significant potential for practical applications in industrial emission-control systems. The CeO_2_ support was excluded in the analysis to decouple the effect of polymers on cobalt.

### 3.2. Structural Characteristics and Surface Properties of the Catalysts

**Figure 3:**
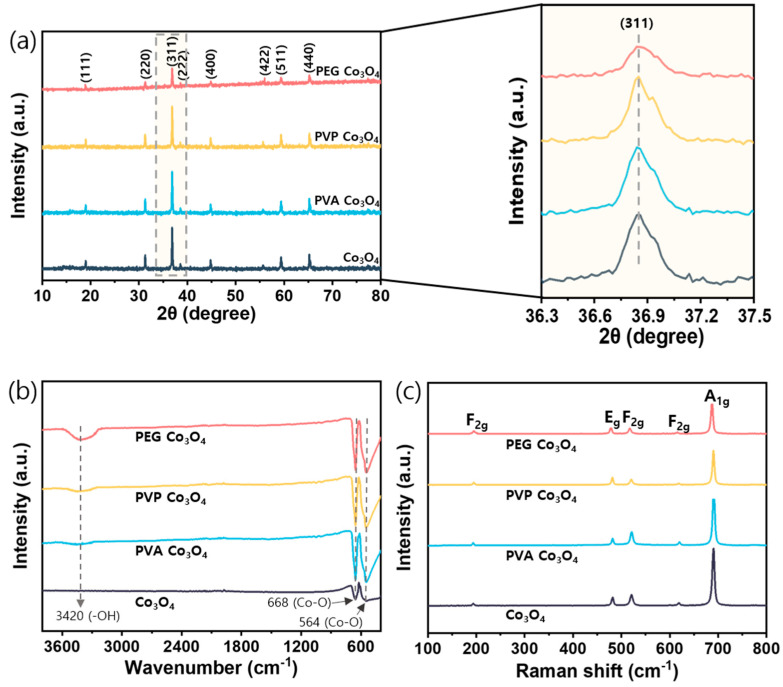
Structural and vibrational characterizations of PEG Co_3_O_4_, PVP Co_3_O_4_, PVA Co_3_O_4_, and pristine Co_3_O_4_ catalysts: (**a**) XRD analysis (**b**) FT-IR spectra (**c**) Raman spectra.

XRD was performed to examine the crystallinities and identify the crystal planes of the synthesized catalysts Figure 3a. The structure of the pristine Co_3_O_4_ catalyst was confirmed by characteristic peaks located at 19.1°, 31.3°, 36.8° and 44.6°, which corresponded to the (111), (220), (311), and (400) planes of Co_3_O_4_, respectively (JCPDS card No. 75-2480) [52]. For the polymer-assisted Co_3_O_4_ catalysts, a decrease in diffraction intensity was observed across all planes. In particular, the intensity of the main (311) crystal plane was significantly reduced in the PEG Co_3_O_4_ sample, indicating a lower crystallinity. This observation is typically associated with smaller crystallite sizes and structural disorder [53,54]. The textural properties of the catalysts were examined using BET analysis to confirm the effects of reduced crystallinity on surface properties Table 1. All polymer-assisted Co_3_O_4_ catalysts exhibited increased BET surface areas compared to the pristine Co_3_O_4_ catalyst. Among them, the PEG Co_3_O_4_ sample showed the highest increase in both surface area (m^2^/g) and pore volume (cm^3^/g). Therefore, the structural modifications induced by PEG increased the accessible surface area, which was beneficial for catalytic reactions. Based on the XRD and BET results, the reduced crystallinity and increased surface area of PEG Co_3_O_4_ likely enhanced the exposure of active sites, thereby improving catalytic activity for N_2_O decomposition [55]. As shown in Table 1, the crystallite sizes determined using the Scherrer equation (Equation (2)) demonstrated that the PEG Co_3_O_4_ catalyst exhibited the smallest crystallite size among the samples. Additionally, the particle size distributions were evaluated from TEM images Figure S4, and the results indicated that the PEG Co_3_O_4_ catalyst exhibited smaller particle sizes compared to the pristine sample [56]. Furthermore, the SAED patterns obtained from TEM analysis confirmed the crystalline nature of the catalysts. Specifically, the pristine Co_3_O_4_, PVA Co_3_O_4_, and PVP Co_3_O_4_ catalysts displayed relatively sharp and distinct diffraction rings, whereas the PEG Co_3_O_4_ exhibited more diffuse and blurred rings, indicating a decrease in crystallinity [57,58].

(2) $$D=\frac{K\lambda }{\beta cos\theta }$$

FT-IR spectroscopy was performed to investigate the vibrational characteristics and chemical bonding of the synthesized catalysts. The FT-IR spectra of the polymer-assisted Co_3_O_4_ catalysts are shown in Figure 3b. These samples exhibited three characteristic absorption bands at approximately 3420, 668, and 564 cm^−1^ [59]. The broad band centered at 3420 cm^−1^ corresponded to the hydroxyl (-OH) stretching vibration. Compared to that of pristine Co_3_O_4_, the enhanced intensity of this hydroxyl band in the PEG Co_3_O_4_ sample suggests an increased surface hydroxyl content, which may be associated with higher numbers of surface defect sites and active oxygen species [60]. The presence of abundant surface hydroxyl groups can enhance catalytic activity by facilitating reactant adsorption and participating in electron transfer processes during redox reactions [61]. Two distinctive bands at 668 and 564 cm^−1^ corresponded to the Co-O stretching vibrations of Co^2+^-O and Co^3+^-O bonds, respectively, confirming the successful formation of a Co_3_O_4_ spinel structure in all synthesized catalysts [59]. When comparing the polymer-assisted catalysts with pristine Co_3_O_4_, significant differences were observed in the intensities of the Co_3_O_4_ stretching bands. For example, the polymer-assisted samples exhibited enhanced and sharper peaks at 668 and 564 cm^−1^, suggesting improved Co_3_O_4_ bonding characteristics. Moreover, the increased intensity and sharpness of these characteristic peaks indicate that the polymer additives may influence the local coordination environment during Co_3_O_4_ formation, potentially contributing to better-defined metal–oxygen bonding in the spinel structure [40,62].

Raman spectroscopy was used to investigate the structural characteristics of the synthesized Co_3_O_4_ catalysts. According to group theory, spinel Co_3_O_4_ exhibits five Raman-active modes: A_1g_, E_g_, and three F_2g_ modes [63]. The Raman spectra of the pristine and polymer-assisted Co_3_O_4_ catalysts are shown in Figure 3c. All samples exhibited the three characteristic Co_3_O_4_ spinel bands, thus indicating the successful formation of a Co_3_O_4_ spinel structure without impurities. For example, the A_1g_ mode appeared at approximately 680 cm^−1^ (octahedral Co^3+^-O_6_ vibration), three F_2g_ modes were located at approximately 190, 515, and 610 cm^−1^ (tetrahedral Co^2+^-O_4_ vibrations), and the E_g_ mode was observed at approximately 478 cm^−1^ [50]. Notably, a gradual decrease in the A_1g_ peak intensity was observed in the polymer-assisted samples in the following order: PEG Co_3_O_4_ < PVP Co_3_O_4_ < PVA Co_3_O_4_ < pristine Co_3_O_4_. This decrease indicates a reduction from Co^3+^ to Co^2+^ during synthesis, because the A_1g_ mode is characteristic of Co^3+^ species in octahedral sites [64]. The reduction process can be attributed to the thermal decomposition of the organic polymers (PVA, PVP, and PEG), which function as mild reducing agents during calcination. Their decomposition releases carbonaceous species that facilitate the partial reduction of Co^3+^ to Co^2+,^ resulting in the formation of oxygen vacancies, which play a key role in enhancing catalytic activity by providing active sites for redox reactions [64,65].

### 3.3. Redox Behavior and Surface Oxygen State Properties

**Figure 4:**
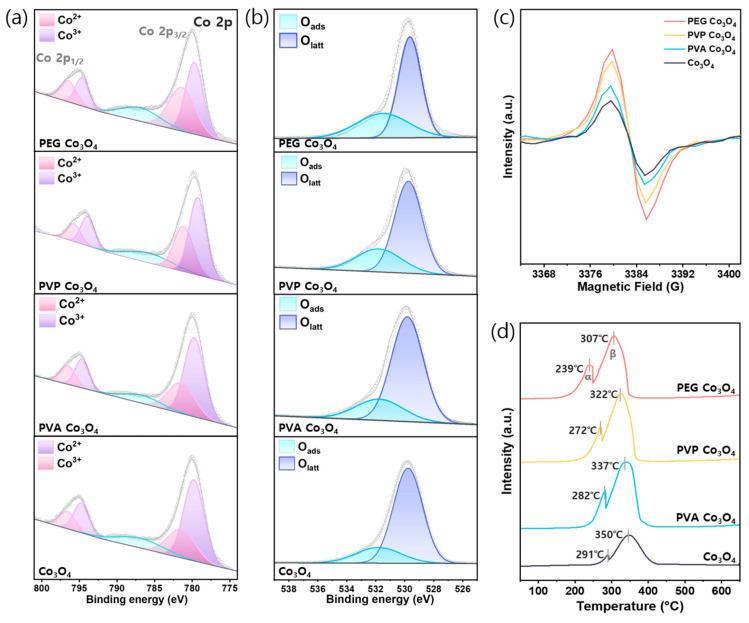
Surface and redox characterizations of PEG Co_3_O_4_, PVP Co_3_O_4_, PVA Co_3_O_4_, and pristine Co_3_O_4_ catalysts: (**a**) Co 2p XPS spectra (**b**) O 1s XPS spectra. (**c**) EPR spectra and (**d**) H_2_-TPR profiles.

As shown in Figure 4a and Table 2, the chemical oxidation states of cobalt were investigated using XPS. The Co 2p spectrum exhibited two primary peaks corresponding to the Co^2+^ and Co^3+^ oxidation states. Particularly, the fitted peaks near 796.4 and 781.3 eV were attributed to Co^2+^ 2p_1/2_ and Co^2+^ 2p_3/2_, respectively. Moreover, the peaks located near 794.7 and 779.6 eV were assigned to Co^3+^ 2p_1/2_ and Co^3+^ 2p_3/2_, respectively [66]. The detailed peak positions and corresponding assignments are summarized in Table S1. According to previous reports, a high number of Co^2+^ ions on the catalyst surface is advantageous for promoting N_2_O decomposition because Co^2+^ can adsorb N_2_O and donate electrons to weaken and cleave N-O bonds [51,67]. The Co 2p spectra revealed that the relative ratio of the Co^2+^ peaks increased in the polymer-assisted Co_3_O_4_ catalysts, particularly in the PEG-assisted sample, suggesting a higher surface concentration of Co^2+^ species. This increased Co^2+^ content may contribute to improved N_2_O decomposition performance because Co^2+^ species serve as the primary active sites in the redox mechanism. In addition, the presence of surface oxygen species plays a crucial role in catalytic reactions. As shown in Figure 4b, the O 1s spectra of the catalysts comprised two main peaks. The peak located at 531.0–531.8 eV was attributed to surface-adsorbed oxygen species (O_ads_), while the peak at 529.1–530.0 eV corresponded to lattice oxygen species (O_latt_). The ratios of O_ads_-to-O_latt_ for all samples are presented in Table 2 [44]. For the polymer-assisted Co_3_O_4_ catalysts, the O_ads_/O_latt_ ratio increased depending on the type of polymer: Co_3_O_4_ < PVA Co_3_O_4_ < PVP Co_3_O_4_ < PEG Co_3_O_4_. This increase was likely due to the formation of additional oxygen vacancies induced by adding the polymer during synthesis. The enhanced formation of surface oxygen vacancies facilitated the generation of reactive oxygen species, which are beneficial for N_2_O decomposition [68]. Overall, the PEG-assisted Co_3_O_4_ catalyst exhibited the highest Co^2+^/(Co^2+^ + Co^3+^) and O_ads_/(O_ads_ + O_latt_) ratios of 0.47 and 0.72, respectively, indicating enhanced oxygen vacancy formation and optimal surface properties for N_2_O decomposition.

EPR spectroscopy is highly sensitive to paramagnetic species, such as unpaired electrons; thus, it was employed to investigate the presence of surface Co^2+^ species and their associated electronic states. As shown in Figure 4c, the pristine and polymer-assisted Co_3_O_4_ catalysts exhibited a characteristic signal at g ≈ 2.003, corresponding to unpaired electrons localized on surface Co^2+^ ions [50]. The signal intensities followed the order: Co_3_O_4_ < PVA Co_3_O_4_ < PVP Co_3_O_4_ < PEG Co_3_O_4_, indicating an increase in the concentration of surface Co^2+^ species. It is likely that the thermal decomposition of polymer additives during calcination facilitated the formation of oxygen vacancies and promoted the generation of Co^2+^ ions on the catalyst surface. These results are consistent with the XPS analysis, further confirming the enhanced surface Co^2+^ species in the polymer-assisted Co_3_O_4_ catalysts [54,69].

The redox behaviors of the synthesized catalysts were investigated using H_2_-TPR analysis Figure 4d. All samples exhibited two distinct hydrogen reduction peaks within the range of 50–700 °C. The first peak (denoted as α, 200–300 °C) corresponded to the reduction of Co^3+^ to Co^2+^, while the second peak (denoted as β, 300–400 °C) corresponded to the reduction of Co^2+^ to metallic cobalt [66]. Compared to pristine Co_3_O_4_, both the α and β peaks of the polymer-assisted Co_3_O_4_ catalysts shifted to lower temperatures. Among them, the PEG-assisted Co_3_O_4_ catalyst exhibited the most pronounced shift, with α and β peaks at 239 and 307 °C, respectively, indicating enhanced surface reducibility and a more facile reduction of cobalt species. These improved redox properties, particularly in PEG-assisted Co_3_O_4_, are expected to play a key role in enhancing catalytic performance for N_2_O decomposition [44,70].

**Figure 5:**
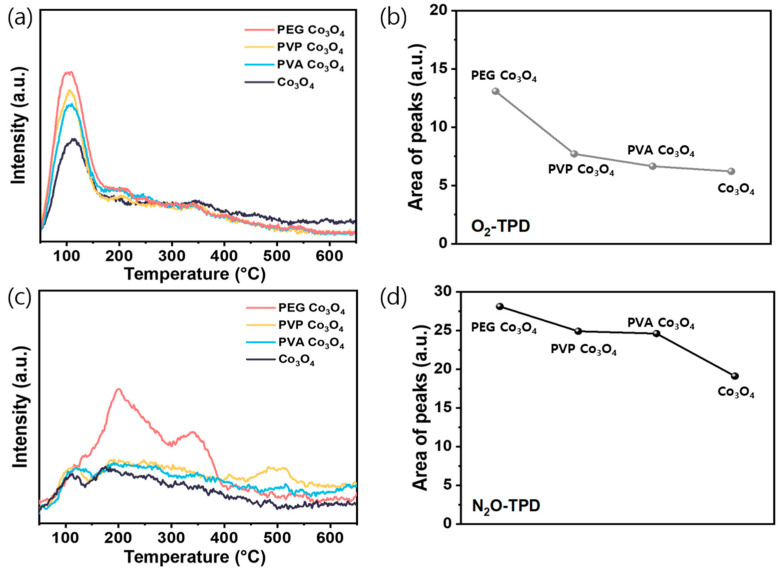
O_2_-TPD and N_2_O-TPD analyses of the PEG Co_3_O_4_, PVP Co_3_O_4_, PVA Co_3_O_4_, and pristine Co_3_O_4_ catalysts: (**a**) O_2_-TPD profile (**b**) Peak areas of O_2_-TPD and (**c**) N_2_O-TPD profile (**d**) Peak areas of N_2_O-TPD.

The TPD of O_2_ and N_2_O was performed to investigate the role of the synthesized catalysts in N_2_O decomposition. First, the O_2_-TPD profiles were recorded over 50–700 °C to examine the surface oxygen reactivity Figure 5a and Table S2. The total desorption amount, as shown in Figure 5b, exhibited the following trend: Co_3_O_4_ < PVA Co_3_O_4_ < PVP Co_3_O_4_ < PEG Co_3_O_4_. Notably, PEG Co_3_O_4_ exhibited a strong and broad desorption peak between 130 and 280 °C, attributed to active surface oxygen species [44]. Because oxygen desorption at ˂450 °C is generally associated with chemisorbed species (O_ads_) [66,67], these results support the XPS findings showing elevated O_ads_/O_latt_ ratios in the polymer-assisted samples. Therefore, the enhanced low-temperature oxygen desorption observed for PEG Co_3_O_4_ indicated a higher density of reactive oxygen species, which could facilitate N_2_O decomposition.

To evaluate the N_2_O activation capacity, N_2_O-TPD analysis was performed. According to previous studies, the TPD signals in this context primarily originate from the desorption of N_2_O or its decomposition products adsorbed on the surface oxygen vacancies [71]. As shown in Figure 5c and Table S2, all samples displayed a primary desorption peak at <450 °C, indicating desorption of surface-adsorbed N_2_O or dissociation products at vacancy sites [72]. This chemisorption-related region reflects the N_2_O adsorption–dissociation capability of the catalysts. Among the samples, PEG Co_3_O_4_ exhibited the highest desorption intensity, followed by PVP Co_3_O_4_, PVA Co_3_O_4_, and pristine Co_3_O_4_. This order was confirmed by the temperature-programmed desorption curves and relative desorption peak areas shown in Figure 5a–d, highlighting that polymer addition enhanced the surface interactions and N_2_O decomposition performance.

### 3.4. Role of Polymer

Herein, polymer-assisted metal oxide catalysts were synthesized using polymers that can regulate the particle size and morphology and induce oxygen vacancies. All polymers coordinated with cobalt precursors during nucleation, suppressing uncontrolled hydrolysis and particle aggregation, while their subsequent thermal decomposition generated reductive intermediates that partially reduced Co^3+^ to Co^2+^ and induced oxygen vacancies [31,32,33,34]. These processes collectively enhanced redox activity and surface defect density, resulting in improved catalytic performance for N_2_O decomposition. Among the three polymers, PVA and PVP primarily influenced structural evolution and surface stabilization during the synthesis process. PVA, containing abundant hydroxyl groups, strongly interacted with hydrated cobalt complexes ([Co(H_2_O)_6_]^2+^) via hydrogen bonding, forming transient core–shell structures that inhibited grain growth and promoted uniform particle dispersion. During calcination, the decomposition of PVA released mildly reducing species, such as polyene fragments, which partially reduced Co^3+^ to Co^2+^ and generated oxygen vacancies through charge compensation associated with lattice oxygen removal. This led to a higher surface Co^2+^ content and enhanced exposure of active sites [34]. PVP, in contrast, contains carbonyl and pyrrolidone nitrogen groups that coordinated with cobalt ions, mitigating excessive hydrolysis and aggregation during precursor formation. Its steric hindrance further restricted crystal growth, thereby stabilizing dispersed Co_3_O_4_ nanoparticles. While PVP induced fewer oxygen vacancies than PVA, it effectively maintained particle uniformity and stabilized surface Co^2+^ species by suppressing excessive oxidation during calcination [73].

**Figure 6:**
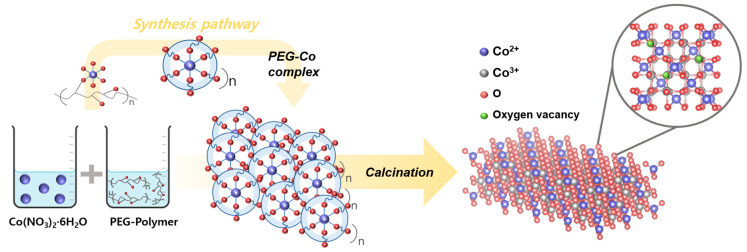
Synthesis pathway of the PEG Co_3_O_4_ catalyst, which promoted Co^2+^ formation.

In particular, PEG contributed to an increased number of surface Co^2+^ species and the formation of oxygen vacancies during the synthesis process. These features are related to structural properties and are closely associated with redox-active surface characteristics. The mechanism responsible for the increased Co^2+^ ratio during the fabrication process is presented in Figure 6 and Equations (3)–(5). After Co(NO_3_)_2_·6H_2_O was dissolved in water to form [Co(H_2_O)_6_]^2+^ complexes [40], the ether oxygen groups in the PEG chain were weakly coordinated to [Co(H_2_O)_6_]^2+^. This resulted in the formation of PEG-[Co^2+^] complexes via hydrogen bonding and dipole-ion interactions. Owing to their flexible chain structure and high hydrophilicity, PEG molecules surrounded the cobalt ions, slowed hydrolysis, and facilitated uniform precipitation [74,75]. After adding NaOH, cobalt hydroxide precipitated and stabilized within the PEG matrix. During calcination, PEG decomposed particularly between 200 and 450 °C, releasing carbonaceous intermediates such as CO, CH_2_O, and aldehydes. These in situ formed reducing species partially reduced Co^3+^ to Co^2+^, while charge compensation was achieved via lattice oxygen removal, resulting in the formation of oxygen vacancies [76]. This trend is consistent with the XPS results, which showed a decreased O_latt_ signal for the PEG Co_3_O_4_ catalyst, indicating the generation of surface oxygen vacancies. These structural and electronic modifications hindered crystal grain growth and resulted in a higher surface area and number of redox-active sites, thereby enhancing the N_2_O adsorption and activation ability of the catalyst.

Co(NO_3_)_2_·6H_2_O → [Co(H_2_O)_6_]^2+^ + 2NO_3_^−^ (3)

[Co(H_2_O)_6_]^2+^ + 2OH^−^ → Co(OH)_2_ + 6H_2_O (4)

6Co(OH)_2_ + O_2_ → 2Co_3_O_4_ + 6H_2_O (5)

The thermal decomposition of PEG occurred in multiple stages. First, dehydration occurred at ˂180 °C, followed by the degradation of hydroxyl and ether groups between 180 and 380 °C and polyether chain scission at approximately 380–500 °C. These stages released reductive fragments that interacted with the cobalt lattice during calcination [49,76]. The resulting oxygen vacancies played a significant role in stabilizing the surface Co^2+^ species.

The Raman spectra showed a weakened A_1g_ mode associated with Co^3+^ to Co^2+^, indicating partial reduction. The XPS Co 2p and O 1s spectra further confirmed the increased Co^2+^/Co^3+^ and O_ads_/O_latt_ ratios in PEG Co_3_O_4_. Moreover, EPR analysis revealed stronger signals at g ≈ 2.003, indicating increased unpaired electrons from oxygen vacancies. These findings were consistent with the improved reducibility observed in the H_2_-TPR results. Moreover, they were supported by the O_2_-TPD and N_2_O-TPD analyses, which indicated the enhanced desorption of surface oxygen species and N_2_O-derived intermediates. In summary, introducing PEG during the Co_3_O_4_ preparation process was highly beneficial for forming catalysts with enriched surface Co^2+^ species and oxygen vacancies, thereby enhancing the catalytic performance in N_2_O decomposition.

## 4. Conclusions

An effective synthesis strategy for Co_3_O_4_ spinel catalysts was developed using polymer (PVA, PVP, or PEG)-assisted methods to enhance the performance of N_2_O decomposition. The addition of these polymers facilitated the formation of Co^2+^-enriched surfaces and abundant oxygen vacancies, which are primary active sites for N_2_O activation. Among the polymers investigated, the PEG-assisted Co_3_O_4_ catalyst exhibited the most pronounced enhancement in terms of surface area, reducibility, and O_ads_/O_latt_ ratio, as demonstrated by XRD, XPS, EPR, TPD, and H_2_-TPR analyses. Notably, the unique characteristics of PEG, such as its abundant hydroxyl groups and flexible molecular chains, enabled strong interactions with cobalt precursors, thus significantly facilitating the preferential formation and stabilization of surface Co^2+^ species. The increased concentration of Co^2+^, which is a key redox-active site in the catalytic cycle, directly contributed to superior N_2_O decomposition performance. Finally, the PEG 5Co_3_O_4_/CeO_2_ sample achieved nearly complete N_2_O conversion (˃99.5%) at 600 °C. Moreover, it maintained a conversion of 97.2% under O_2_ co-feeding conditions, thus outperforming catalysts synthesized without polymer additives. This study demonstrates that PEG-assisted synthesis can effectively modulate the surface chemistry of Co_3_O_4_ by enriching catalytically active Co^2+^ sites, thereby significantly enhancing N_2_O decomposition activity. Thus, polymer-assisted spinel catalysts can be a practical approach for improving N_2_O decomposition efficiency.

## Figures and Tables

**Table 1:** BET surface areas, pore volumes and crystallite size of PEG Co_3_O_4_, PVP Co_3_O_4_, PVA Co_3_O_4_, and pristine Co_3_O_4_.

Sample	BET Surface Area (m^2^/g)	Pore Volume (cm^3^/g)	Pore Size (nm)	Crystallite Size (nm)
PEG Co_3_O_4_	35	20	17	15.64
PVP Co_3_O_4_	25	12	12	16.59
PVA Co_3_O_4_	24	12	11	16.82
Co_3_O_4_	18	10	10	17.25

**Table 2:** XPS results of the PEG Co_3_O_4_, PVP Co_3_O_4_, PVA Co_3_O_4_, and pristine Co_3_O_4_ catalysts.

Sample	Surface Information	
	Co^2+^/(Co^2+^ + Co^3+^)	O_ads_/(O_ads_ + O_latt_)
PEG Co_3_O_4_	0.47	0.72
PVP Co_3_O_4_	0.43	0.64
PVA Co_3_O_4_	0.35	0.50
Co_3_O_4_	0.30	0.41

## Data Availability

The original contributions presented in this study are included in the article/Supplementary Material. Further inquiries can be directed to the corresponding authors.

## Supplementary Materials

The following supporting information can be downloaded at https://www.mdpi.com/article/10.3390/nano15211642/s1, Figure S1. A scheme of materials synthesis. Figure S2. N2O decomposition activity of PEG, PVP, and PVA-assisted Co_3_O_4_ catalysts and pristine 5Co_3_O_4_ under 5000 ppm N2O, N2 balance, GHSV 60,000 h^−1^, in the temperature range of 350–650 °C. Figure S3. Catalytic performance and kinetic analysis of all sample catalysts for N2O decomposition. (a) N2O conversion as function of reaction temperature (b) Reaction rates at various temperature (c) Arrhenius plot (d) Activation energy (Ea). Reaction condition: 5000 ppm N2O with N2 balance, GHSV=1,000,000 h^−1^. Figure S4. TEM images and SAED patterns of the catalysts. (a) and (b) Pristine Co_3_O_4_, (c) and (d) PVA Co_3_O_4_, (e) and (f) PVP Co_3_O_4_, (g) and (h) PEG Co_3_O_4_. Table S1. XPS peaks data of the PEG Co_3_O_4_, PVP Co_3_O_4_, PVA Co_3_O_4_, and pristine Co_3_O_4_ catalysts. Table S2. Peak area data from the O2-TPD and N2O-TPD analyses.
