# Supplementary material for: Polymer-Assisted Synthesis of Co3O4 Spinel Catalysts with Enhanced Surface Co2+ Ions for N2O Decomposition
Source: Nanomaterials (Basel). 2025 Oct 28;15(21):1642. doi: 10.3390/nano15211642 (PMC12608398; doi:10.3390/nano15211642)
Supplement: Supplementary file 1 [file nanomaterials-15-01642-s001.zip › nanomaterials-3905971-supplementary.pdf]

Supporting Information

Polymer-Assisted Synthesis of Co_3O_4 Spinel Catalysts with Enhanced Surface Co^{2+} Ions for N_2O Decomposition

Nahea Kim[1,2], Su-Jin Kim[1], Sang-Hyeok Seo[1], Myeung-Jin Lee[1], Bora Jeong[1], Hong-Dae Kim[1], Tae Won Nam[2, *], and Bora Ye[1, *]

[1]Ulsan Technology Application Division, Korea Institute of Industrial Technology, Ulsan 44413, Republic of Korea; yebora@kitech.re.kr.

[2]Department of Materials Science and Engineering, Pusan National University, Busan 46241, Republic of Korea; namtaewon@pusan.ac.kr.

1*. Bora Ye. Tel.: +82)52-980-6644; Fax: +82)52-980-6644, E-mail: yebora@kitech.re.kr.
2*. Tae Won Nam. Tel.: +82)51-510-2445 E-mail: namtaewon@pusan.ac.kr.

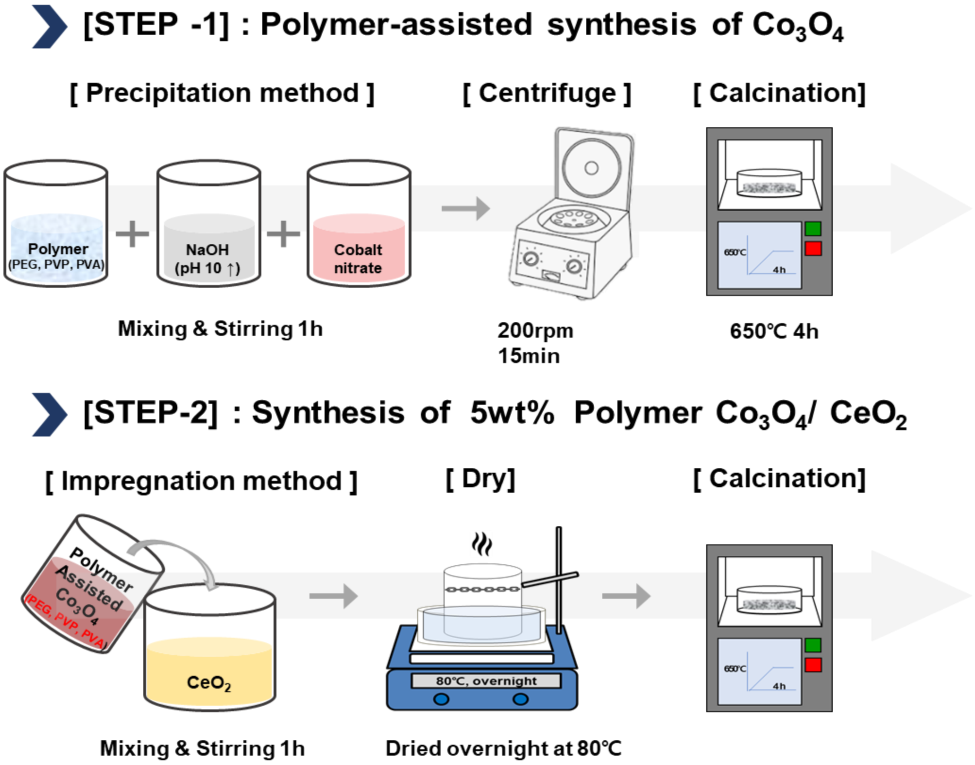

Figure S1. Scheme of materials synthesis

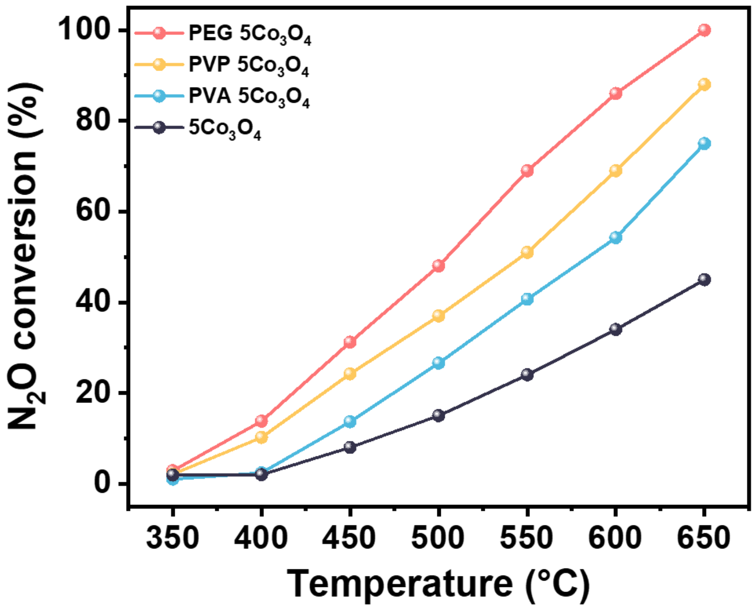

Figure S2. N_2O decomposition activity of PEG, PVP, and PVA-assisted Co_3O_4 catalysts and pristine $5Co_3O_4$ under 5000 ppm N_2O, N_2 balance, GHSV 60,000 h^{-1}, in the temperature range of 350–650 °C.

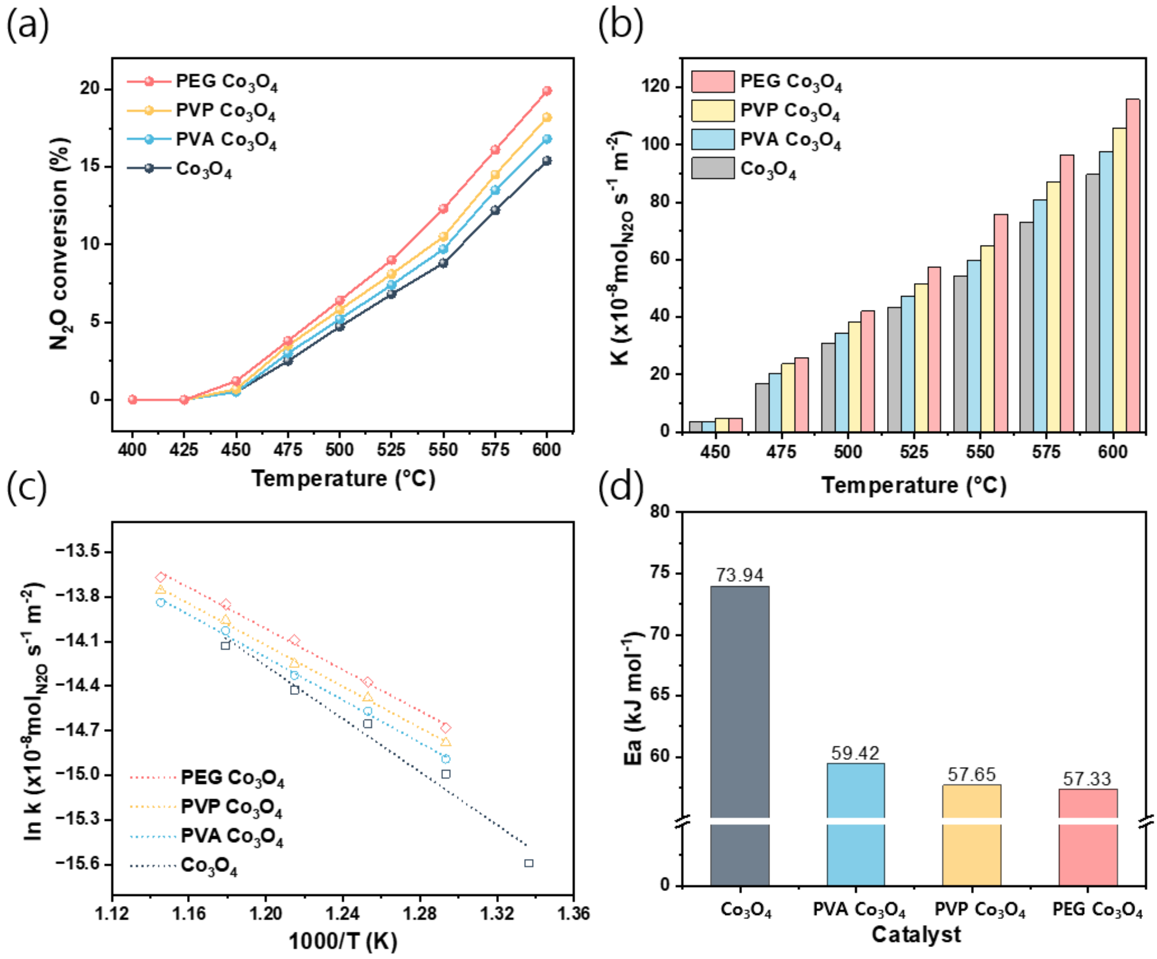

Figure S3. Catalytic performance and kinetic analysis of all sample catalysts for N_2O decomposition. a) N_2O conversion as function of reaction temperature b) Reaction rates at various temperature c) Arrhenius plot d) Activation energy (E_a). Reaction condition: 5000 ppm N_2O with N_2 balance, GHSV=1,000,000 h^{-1}.

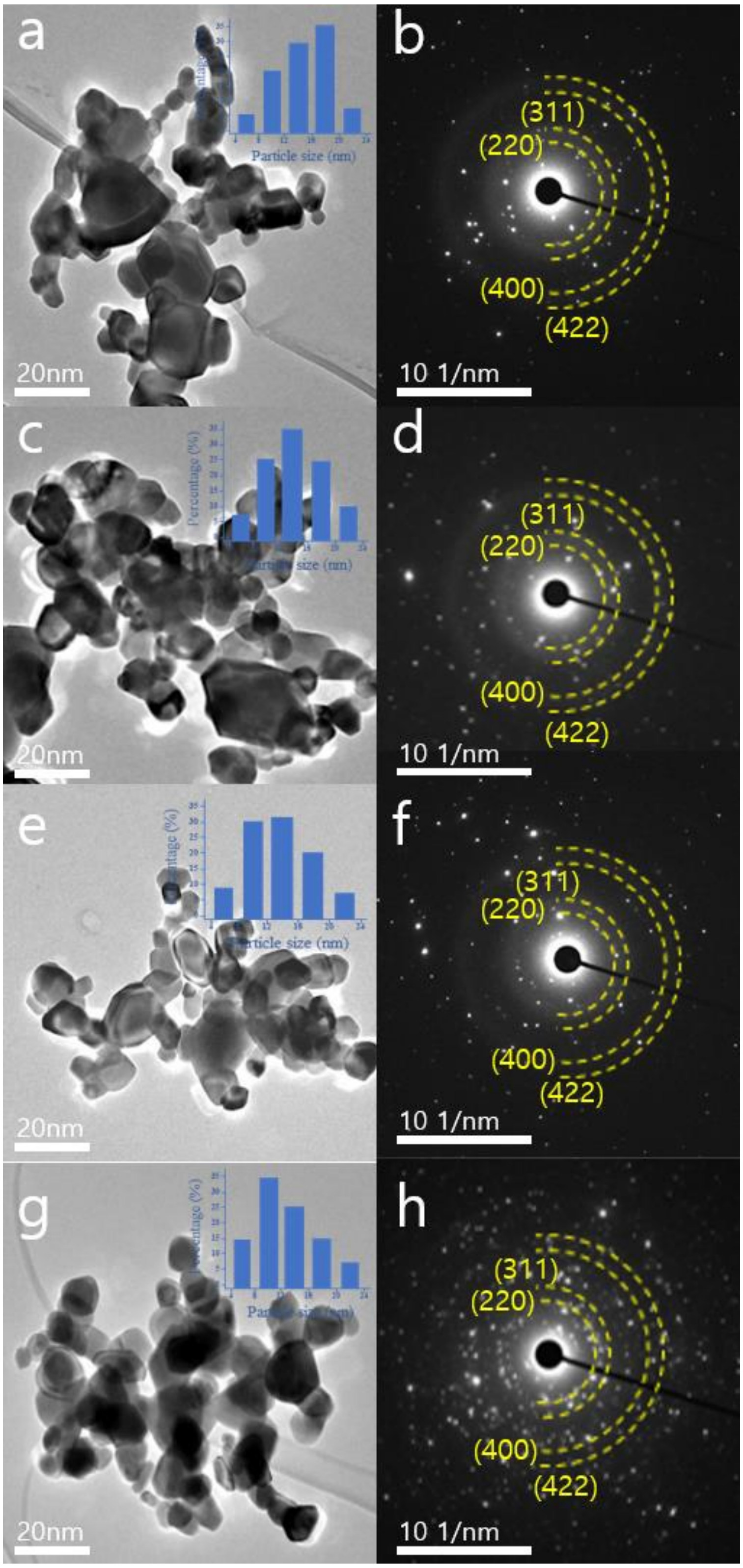

Figure S4. TEM images and SAED patterns of the catalysts.
(a) and (b) Pristine Co_3O_4, (c) and (d) PVA Co_3O_4, (e) and (f) PVP Co_3O_4, (g) and (h) PEG Co_3O_4.

Table S1. XPS peaks data of the PEG Co_3O_4, PVP Co_3O_4, PVA Co_3O_4, and pristine Co_3O_4 catalysts.

Binding energy (eV)						
Sample	$Co^{2+}{}_{2p1/2}$	$Co^{3+}{}_{2p1/2}$	$Co^{2+}{}_{2p3/2}$	$Co^{3+}{}_{2p3/2}$	O_{ads}	O_{latt}
PEG Co_3O_4	796.5	794.7	781.4	779.7	531.5	529.9
PVP Co_3O_4	796.6	794.7	781.3	779.6	531.6	529.7
PVA Co_3O_4	796.5	794.6	781.4	779.7	531.8	529.8
Co_3O_4	796.5	794.6	781.3	779.7	531.8	529.7

Table S2. Peak area data from the O_2-TPD and N_2O-TPD analyses.

Sample	O_2-TPD		N_2O-TPD	
	Temperature (℃)	Area of peaks (×10^{-10})	Temperature (℃)	Area of peaks (×10^{-10})
PEG Co_3O_4	175, 299	13.08	48	28.1
PVP Co_3O_4	97, 165, 443	7.71	59	24.9
PVA Co_3O_4	103, 166	6.65	58	24.6
Co_3O_4	95, 150	6.21	65	19.1
